# Sex differences in levodopa pharmacokinetics in early Parkinson’s disease: implications on levodopa-related complications

**DOI:** 10.3389/fphar.2026.1748932

**Published:** 2026-04-07

**Authors:** Valeria Conti, Emanuela De Bellis, Graziamaria Corbi, Bruno Charlier, Viviana Izzo, Maria Claudia Russillo, Marianna Amboni, Marina Picillo, Cesa Lorella Maria Scaglione, Ilaria Cani, Calogero Edoardo Cicero, Alessandra Nicoletti, Andrea Soricelli, Paolo Barone, Amelia Filippelli, Maria Teresa Pellecchia

**Affiliations:** 1 Department of Medicine, Surgery, and Dentistry, Scuola Medica Salernitana, University of Salerno, Baronissi, Italy; 2 Clinical Pharmacology Unit, San Giovanni di Dio e Ruggi d’Aragona University Hospital, Salerno, Italy; 3 PhD School “Clinical and Translational Oncology (CTO)”, Scuola Superiore Meridionale, University of Naples “Federico II”, Naples, Italy; 4 Department of Translational Medical Sciences, University of Naples “Federico II”, Naples, Italy; 5 Postgraduate School of Clinical Pharmacology and Toxicology, University of Salerno, Baronissi (SA), Italy; 6 Neuroscience Section, Department of Medicine, Surgery and Dentistry “Scuola Medica Salernitana”, University of Salerno, Baronissi, Italy; 7 Neurological Clinic, San Giovanni di Dio e Ruggi d’Aragona University Hospital, Salerno, Italy; 8 IRCCS “Istituto delle Scienze Neurologiche di Bologna”, UOC Neurological Clinic, Bellaria Hospital, Bologna, Italy; 9 Neurologic Unit, AOU “Policlinico-San Marco”, Department of Medical, Surgical Sciences and Advanced Technologies, GF Ingrassia, University of Catania, Catania, Italy; 10 IRCCS SYNLAB SDN, Naples, Italy

**Keywords:** dyskinesia, levodopa, motor and non-motor fluctuations, Parkinson’s disease, sex, wearing off

## Abstract

**Introduction:**

Women with Parkinson’s Disease (PD) have higher plasma exposure to levodopa and are particularly prone to developing complications during chronic levodopa therapy. However, there are no longitudinal studies focusing on differences in levodopa pharmacokinetics and their correlation with clinical outcomes. This multicenter longitudinal study aimed to investigate sex-related differences in levodopa pharmacokinetics in levodopa-naïve PD patients, and to evaluate relationships with levodopa-related complications.

**Methods:**

After a single dose of levodopa/DOPA-decarboxylase inhibitor, blood samples were collected at baseline and at two-year follow-up to measure pharmacokinetic parameters using UHPLC-MS. Clinical assessment included wearing-off Questionnaire and MDS-UPDRS scale. Multiple linear regression analyses were performed to identify predictors of pharmacokinetic parameters and levodopa-related complications.

**Results and Discussion:**

The study population consisted of 28 PD patients (18 men, 10 women) followed for 2 years from the start of levodopa therapy. No differences were found between the sexes in clinical characteristics, daily levodopa-dosage, and use of other antiparkinsonian drugs. AUC and Cmax were higher in females than in males (p < 0.001), and sex influenced both of these parameters regardless of whether pharmacokinetic analysis was performed at baseline or at follow-up. Female sex was the best predictor of AUC and Cmax. DYS was found in 20% of females but in no males, and 90% of females showed wearing-off compared to 50% of males (p = 0.022). In females but not in males, the presence of wearing -off was correlated with AUC and Cmax at baseline and after 2 years of treatment. Compared to men, women with PD showed higher plasma levodopa levels since the initial administration and after chronic treatment. Measuring exposure to levodopa may be useful for optimising levodopa dosage since the start of treatment, thus preventing levodopa-related complications.

## 1 Introduction

Although levodopa (LD) is the gold standard for the treatment of Parkinson’s disease (PD) due to its great efficacy, its long-term use is closely related to motor and non-motor fluctuations (MNMF) and dyskinesias (DYS) (Müller and Russ, 2006; Pahwa and Lyons, 2009).

Factors related to the disease (e.g., duration and severity), the drug (dose and formulation), and the patient (e.g., age at disease onset, body weight, and sex) may influence the clinical outcome (Zappia et al., 2002).

Female sex is recognized as an important risk factor for the development of MNMF (Hassin-Baer et al., 2011; Picillo et al., 2016; Eusebi et al., 2018). DYS have been reported, particularly in women with low body weight, even following the administration of a low dosage of LD (100 mg or less) (Martinez-Ramirez et al., 2014).

Furthermore, pulsatile dopaminergic stimulation, caused by multiple oral LD doses, may be a primary cause of wearing-off (WO) and DYS (Olanow et al., 2006), especially during disease progression, when dopaminergic terminals lose their ability to counteract fluctuations in plasma LD levels. Consequently, in PD patients, plasma LD levels may rise and fall in a pulsatile manner, leading to motor complications (Shiraishi et al., 2020). Several studies have reported higher LD Area Under the Curve (AUC) and maximum Concentration (Cmax) in women than in men, supporting sex as the most relevant variable explaining the relationship between pharmacokinetics (PK) and LD motor complications (Contin et al., 2022; Nishikawa et al., 2020).

Based on this evidence, assessing the potential relationship between PD patient characteristics and LD pharmacokinetics becomes crucial.

In a recent study, we found sex differences in plasma LD concentrations in PD patients taking their first-ever dose of LD. Compared to men, women showed higher plasma LD concentrations measured over a time interval of 20–260 min, and higher AUC (2.584 ± 1.499 in women vs. 1.127 ± 0.678 in men) and Cmax (2405.09 ± 1732.54 in women vs. 909.89 ± 638.21 in men) (Conti et al., 2022).

The present investigation aimed to compare plasma LD concentrations and pharmacokinetic parameters between sexes in the same population, which was followed for 2 years after starting LD treatment, and to assess the potential correlation with LD-related fluctuations and DYS.

## 2 Materials and methods

The study population consisted of a subset of patients (28 out of 35) diagnosed with PD according to MDS clinical diagnostic criteria^13^, enrolled in a multicenter longitudinal study approved by the Ethics Committees of the participating centers (n.4_r.p.s.o./2019 for the Coordinating Center of Salerno).

The patients were followed for 2 years after starting LD treatment at the Center for Neurodegenerative Diseases (CEMAND), Department of Medicine, Surgery and Dentistry “Scuola Medica Salernitana,” University of Salerno, Italy; Movement Disorders Centre, Hermitage-Capodimonte, Naples; Dipartimento “G.F. Ingrassia, ”Neuroscience Unit- University of Catania, Italy; I.R.C.C.S.-“Istituto di Scienze Neurologiche and DIBINEM”- “Alma Mater Studiorum”–University of Bologna, Italy.

Of the 35 patients originally enrolled, 10 women and 18 men completed the 2-year Follow-up and consented to undergo PK assessment. Specifically, 4 women and 1 man were lost to Follow-up due to restrictions on returning to the hospital during the COVID pandemic, and 2 women completed the 2-year clinical Follow-up but did not consent to PK assessment.

Development of DYS at Follow-up was defined by Movement Disorders Society-Unified Parkinson’s disease rating scale-part IV (MDS-UPDRS-IV) score >0 on item 4.1 (Postuma et al., 2015). Development of MNMF at Follow-up was defined as a score ≥2 in the 19-item Wearing-Off Questionnaire (WOQ-19) (Pellecchia et al., 2025). Baseline and Follow-up MDS-UPDRS III were similar between men and women (26.2±9.3 vs. 26.4±9.9 at baseline; 30.3±8.7 vs. 30.7±8.5 at Follow-up, respectively).

PK analysis was centralized at the Clinical Pharmacology Unit, University Hospital of Salerno. Venous blood samples were collected in EDTA-2Na, in fasting conditions at the visit, through an indwelling catheter before and 20 (T1), 40 (T2), 60 (T3), 80 (T4), 125 (T5), 170 (T6), 215 (T7), and 260 (T8) min after first-morning intake of a single dose of LD/benserazide (100/25 mg) formulation. At the end of the PK test, all patients continued with their usual daily dosage regimen consisting of their formulation (LD/benserazide, 100/25 mg or 200/50 mg or LD/carbidopa, 100/25 mg or 250/25 mg).

LD concentrations were measured by UHPLC-MS as previously reported (Conti et al., 2022). The PK parameters, AUC, Cmax, time to reach Cmax (Tmax), and half-life (t1/2) were calculated using the R 3.5.1 version (Conti et al., 2022) and Prism 8.0.1 version (GraphPad Software, Inc., La Jolla, CA, United States), considering a non-compartmental study model. Given the possibility of pre-dose concentrations other than zero at Follow-up, the concentration of each subject at T0 was subtracted at each time point according to the formula Ccorr(t) = C(t)−C (T0). The values of LD concentrations and LD PK parameters measured at baseline were compared with those measured at Follow-up.

### 2.1 Statistical analysis

Continuous variables (normally distributed) were expressed as mean ± standard deviation (SD) and compared using (Welch t-test). Continuous variables not normally distributed were expressed as median and compared using the Mood’s median test. Categorical variables were expressed as counts and percentages and compared using the Fisher’s exact test. A p-value <0.05 was considered statistically significant.

To determine the effects of time (time of the PK test) performed at baseline or Follow-up on LD concentrations at each time point (T0-T8) and on PK parameters, a mixed-effect model (with as dependent variables: the LD concentrations or the PK parameters; as random effect: intercept for subject; as fixed effects: sex, time of the PK test, and interactions) was used. To account for the pairwise comparisons, adjusted p-values were calculated using the Bonferroni method. Differences were considered statistically significant if the adjusted p-value was <0.05. Moreover, to define the best predictors of AUC, Cmax, Tmax, and t1/2, multivariate analyses were performed using PK parameters as dependent variables, and age, sex, and either body weight (BW) or body mass index (BMI) as independent variables. All values were expressed as mean and standard deviation (SD). A p < 0.05 was considered statistically significant. Statistical analysis was performed using STATA 16 version, and IBM SPSS 30.0.0.0.

## 3 Results

Plasma LD concentrations were measured in 28 PD patients (18 men and 10 women). The study population appeared homogeneous for age and disease duration. No differences were found between men and women in BW, BMI, daily LD dosage, and DOPA decarboxylase inhibitor (DDCI). Comorbidities, the use of monoamine oxidase-B inhibitors (iMAOB), and dopamine agonists (DA) did not differ between sexes. The main demographic, biometric, and clinical characteristics of the study population are shown in Table 1.

**TABLE 1:** Patient characteristics of men and women enrolled in the study after 2 years of LD/DDCI treatment.

Patient characteristics	Men (n = 18)	Women (n = 10)	P-value
Mean ± SD age (years)	64.47 ± 7.91	61.33 ± 12.11	0.496
BW median value (Kg)	75.50	70.0	0.430
BMI median value (Kg/m2)	26.09	28.34	0.115
Duration of the disease (months)	48.27 ± 18.49	60.55 ± 29.26	0.193
Mean ± SD daily LD dosage (mg/day)	350 ± 169.77	360 ± 172.88	0.884
DDCI type (n.,%)			
Carbidopa	12 (66.7%)	5 (50%)	0.444
Benserazide	6 (33.3%)	5 (50%)	0.444
Antiparkinsonian drugs other than levodopa (n.,%)			
DA	1 (5.55%)	1 (10%)	1.000
IMAO-B	1 (5.55%)	1 (10%)	1.000
DA and iMAO-B	14 (77.7%)	5 (50%)	0.210
Comorbidities (n.,%)			
Arterial hypertension	11 (61.1%)	5 (50%)	1.000
Hypercholesterolemia	4 (22.2%)	1 (10%)	0.636
Chronic gastritis	3 (16.7%)	0 (0%)	0.529
Type II diabetes	2 (11.1%)	0 (0%)	0.538

Abbreviations: BW, body weight; BMI, body mass index; DA, dopamine agonists; DDCI, DOPA, decarboxylase inhibitor; iMAO, Monoamino Oxidase-B inhibitors.

### 3.1 Plasma LD concentrations at follow-up compared to baseline in the entire study population and by sex

Our first analysis aimed to assess changes at Follow-up as compared to baseline in the LD plasma concentrations at each time point. Table 2 shows the mean value ± SD and confidence intervals (CI) of plasma LD concentrations measured at baseline and Follow-up from T0 (before drug intake) to T8.

**TABLE 2:** LD plasma concentrations (ng/mL) at baseline and Follow-up from T0 (at the time of drug intake) to T8 by sex, and Type III Tests of Fixed Effects.

Time points	Time of the PK test	Male		95% confidence interval		Female		95% confidence interval	
		Mean	SD	Lower bound	Upper Bound	Mean	SD	Lower bound	Upper bound
T0	Baseline	0.000	0.000	---	---	0.000	0.000	---	---
​	Follow-up	72.976	34.993	−14.418	160.370	90.706	123.844	−0.077	181.490
T1	Baseline	288.841	271.626	−1108.776	1686.457	1041.515	987.178	−410.190	2493.219
​	Follow-up	612.416	667.249	−789.918	2014.750	2367.242	1582.560	915.537	3818.946
T2	Baseline	658.922	481.881	−1401.819	2719.662	2797.516	2024.843	657.024	4938.008
​	Follow-up	767.262	791.987	−1300.43	2834.959	2731.485	1981.203	590.993	4871.977
T3	Baseline	628.008	557.702	−482.919	1738.935	1522.880	818.578	368.865	2676.895
​	Follow-up	975.427	650.673	−135.500	2086.354	1588.641	895.669	434.626	2742.656
T4	Baseline	415.633	310.767	−401.072	1232.338	926.860	481.9177	78.470	1775.233
​	Follow-up	910.038	613.545	93.333	1726.743	1340.762	648.6729	492.381	2189.144
T5	Baseline	314.529	204.540	−277.685	906.743	719.869	419.713	104.686	1335.053
​	Follow-up	614.917	479.292	22.703	1207.131	901.220	352.666	286.036	1516.403
T6	Baseline	225.653	171.131	−200.337	651.644	457.834	256.579	15.321	900.347
​	Follow-up	486.690	372.116	60.700	912.681	608.546	196.418	166.033	1051.059
T7	Baseline	150.404	100.355	−139.787	440.596	418.749	155.775	114.195	723.304
​	Follow-up	400.665	263.028	110.474	690.856	437.703	140.371	136.281	739.125
T8	Baseline	113.655	84.242	−144.958	372.269	213.389	94.795	−57.055	483.834
​	Follow-up	353.082	244.092	94.469	611.696	332.072	131.454	64.397	599.747

Time points	​	Time of the PK test	Sex	Time of the PK test * sex
T0	F	25.163	0.295	0.295
​	Sig	<0.001	0.589	0.589
T1	F	9.905	22.895	3.657
​	Sig	0.003	<0.001	0.062
T2	F	0.003	28.194	0.051
​	Sig	0.957	<0.001	0.822
T3	F	0.992	13.219	0.461
​	Sig	0.324	<0.001	0.501
T4	F	8.873	9.542	0.070
​	Sig	0.005	0.003	0.793
T5	F	4.747	9.784	0.290
​	Sig	0.035	0.003	0.593
T6	F	6.702	4.955	0.481
​	Sig	0.013	0.031	0.491
T7	F	5.957	7.665	4.397
​	Sig	0.019	0.008	0.042
T8	F	13.156	0.636	1.496
​	Sig	<0.001	0.430	0.228

The mixed-effects model was built by using as dependent variables: the LD, concentrations; as random effect: intercept for subject; as fixed effects: sex, time of the PK, test, and interactions. The p-value was denoted as “Sig”. A p < 0.05 was considered statistically significant.

Abbreviations: F, F-statistic; PK, pharmacokinetics; SD, standard deviation; Sig., Significance (p-value).

The effect of time of the PK test (baseline vs. Follow-up) was statistically significant at each time point with the exception of T2 and T3 (Table 2).

**FIGURE 1:**
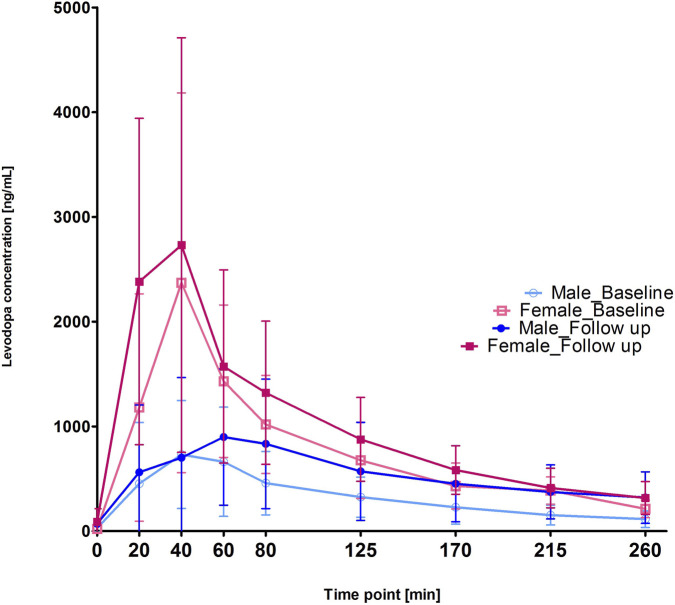
Differences in plasma levodopa (LD) concentrations between men and women at baseline and at Follow-up.

The effect of sex was statistically significant, especially between T1 and T2 (p < 0.001), remained highly statistically significant at T3 (p < 0.001) when the curve drastically decreased in females but not in males, and disappeared only at the end (T8). The interaction between time and sex was not statistically significant, indicating that the effects of time of the PK test and sex were independent of each other (Table 2). As shown in Figure 1, LD concentrations, which were higher in women than in males at baseline, remained higher in females than in males at Follow-up (Figure 1). Notably, from T2 (40 min) to T3 (60 min), LD concentrations decreased dramatically with a Δ% of −42% in females, while they increased in males (Δ% of 28%) and then decreased with a Δ% of −7% between T3 (60 min) and T4 (80 min). The slope calculated between 40 and 60 min was of −57.94 ng·mL^−1^·min^−1^ in females and +9.26 ng·mL^−1^·min^−1^ in males (Figure 1). The difference between sexes was statistically significant (Welch t-test as t (11) = t (3,2533), p = 0.0078).

### 3.2 PK parameters at follow-up compared to baseline in the entire study population and by sex

**FIGURE 2:**
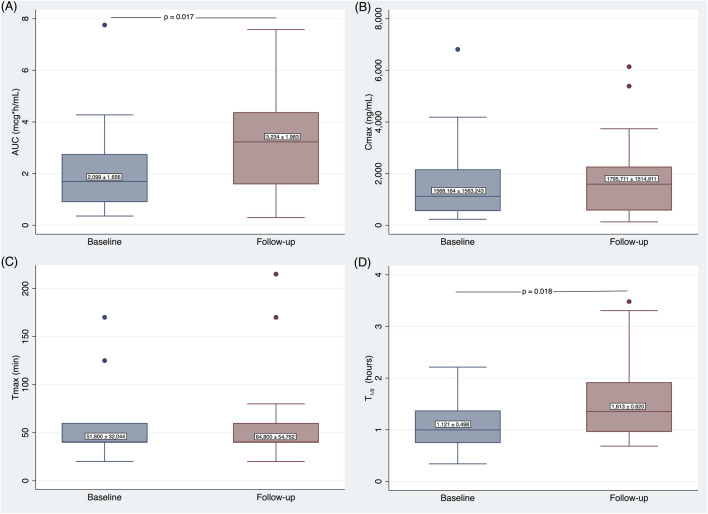
Differences in AUC **(A)**, Cmax **(B)**, Tmax **(C)**, and T_1/2_ **(D)** at Follow-up compared to baseline. AUC, Area Under the Curve; C_max_, Maximum Concentration; T_max_, Time to reach C_max_; T_1/2_, half-life.

The effect of time of the PK test on AUC was statistically significant (p = 0.017), indicating a relevant difference between baseline and Follow-up. Specifically, the mean AUC at baseline was 1.116 mcg*h/mL lower than at Follow-up (p = 0.017) (Figures 2A-D).

The effect of sex on AUC was highly significant (p < 0.001), showing a relevant difference in AUC between sexes. Notably, the mean AUC of females was significantly higher than that of men by 2.130 mcg*h/mL. The interaction between time of the PK test and sex was not statistically significant, indicating that the effects of time of the PK test and sex on AUC were independent of each other (Table 3).

**TABLE 3:** PK parameters at baseline and Follow-up by sex and Type III tests of Fixed effects.

PK parameters	Time of the PK test	Male		95% Confidence interval		Female		95% Confidence interval	
		Mean	SD	Lower bound	Upper bound	Mean	SD	Lower bound	Upper bound
AUC	Baseline	1.306	0.795	−1.111	3.723	3.508	1.884	0.998	6.019
mcg*h/mL	Follow-up	2.494	1.510	0.077	4.911	4.551	2.116	2.040	7.062
Cmax	Baseline	829.725	588.311	−1135.984	2795.434	2880.944	1911.239	838.994	4922.895
ng/mL	Follow-up	1157.085	736.733	−808.624	3122.793	2931.048	1897.724	889.098	4972.999
Tmax	Baseline	49.063	25.181	−21.595	119.720	56.667	43.012	−16.732	130.065
min	Follow-up	74.375	57.933	3.717	145.033	47.778	46.845	−25.621	121.176
T1/2	Baseline	1.201	0.475	0.137	2.265	0.980	0.535	−0.124	2.085
h	Follow-up	1.734	0.875	0.658	2.810	1.437	0.747	0.333	2.542

​	​	Time of the PK test	Sex	Time of the PK test *Sex
AUC	F	6.113	22.282	0.026
mcg*h/mL	Sig	0.017	<0.001	0.872
Cmax	F	0.265	27.164	0.143
ng/mL	Sig	0.61	<0.001	0.707
Tmax	F	0.388	0.518	1.681
min	Sig	0.537	0.475	0.201
T1/2	F	6.025	1.648	0.035
h	Sig	0.018	0.206	0.852

The mixed-effects model was built by using as dependent variables: the PK, parameters; as random effect: intercept for subject; as fixed effects: sex, time of the PK, test, and interactions. The p-value was denoted as “Sig”. A p < 0.05 was considered statistically significant.

Abbreviations: AUC, area under the curve; C_max_, Maximum Concentration; .F, F-statistic; PK, pharmacokinetics; SD, standard deviation; Sig., Significance (p-value); T_max_, Time to reach C_max_; T_1/2_, half-life.

The effect of time of the PK test on Cmax was not statistically significant, indicating no difference between baseline and Follow-up. The effect of sex on Cmax was highly significant (p < 0.001), showing a relevant difference in Cmax between sexes. Specifically, the mean Cmax of females was significantly higher (p < 0.001) than that of men by 366.966 ng/mL. The interaction between time of the PK test and sex was not statistically significant, indicating that the effects of time of the PK test and sex on Cmax were independent of each other (Table 3).

The effect of time of the PK test on Tmax was not statistically significant, indicating no difference between baseline and Follow-up. The effect of sex on Tmax was also not statistically significant, showing no differences between sexes. The interaction between time and sex was not statistically significant, suggesting that the effect of time of the PK test on Tmax did not significantly differ between the sexes (Table 3).

The effect of time of the PK test on t1/2 was statistically significant (p = 0.018), indicating a difference between baseline and Follow-up. Specifically, the mean t1/2 at baseline was 0.495 h lower than at Follow-up. The effect of sex on t1/2 was not significant, indicating no difference between sexes. The interaction between time of the PK test and sex was not statistically significant, suggesting that the effect of time on t1/2 did not significantly differ between the sexes (Table 3).

Different regression analyses were performed to identify potential predictors of the LD PK at Follow-up using each PK parameter as the dependent variable, and age, sex, and BMI or BW as independent variables.

The fitted regression model for AUC was: predicted AUC at Follow-up = 1.276 + (0.043 x age) - (0.054 x BMI) + (2.18 x females). The overall regression was statistically significant (*r* ^2^ = 0.308, F (3, 21) = 3.11, p = 0.048). The best predictor of AUC at Follow-up was represented by female sex (beta = 2.176, 95% CI 0.632 3.720, p = 0.008).

Considering BW rather than BMI, the fitted regression model for AUC was: predicted AUC at Follow-up = - 1.986 + (0.048 x age) + (0.019 x BW) + (2.31 x females). The overall regression was statistically significant (*r* ^2^ = 0.3062, F (3, 21) = 3.09, p = 0.049). The best predictor of AUC at Follow-up was represented by female sex (beta = 2.307, 95% CI 0.597 4.017, p = 0.011).

The fitted regression model for Cmax was: predicted Cmax at Follow-up = −1785.772 + (37.383 x age) + (23.046 x BMI) + (1821.639 x females). The overall regression was statistically significant (*r* ^2^ = 0.374, F (3, 21) = 4.18, p = 0.018). As for AUC, the best predictor of Cmax at Follow-up was represented by female sex (beta = 1821.639, % CI 700.290 2942.988, p = 0.003).

Considering BW rather than BMI, the fitted regression model for Cmax was: predicted Cmax at Follow-up = −2093.153 + (37.155 x age) + (11.850 x BW) + (1944.078 x females). The overall regression was statistically significant (*r* ^2^ = 0.376, F (3, 21) = 4.22, p = 0.017). The best predictor of Cmax at Follow-up was represented by female sex (beta = 1944.078, 95% CI 705.647 3182.509, p = 0.004).

The fitted regression model for Tmax was: predicted Tmax at Follow-up = 180.364 + (0.277 x age) – (4.506 x BMI) – (22.536 x females). The overall regression was not statistically significant (*r* ^2^ = 0.142, F (3, 21) = 1.16, p = 0.349). No predictors of Tmax at Follow-up were found.

Considering BW rather than BMI, the fitted regression model for Tmax was: predicted Tmax at Follow-up = 173.149 + (0.393 x age) – (1.534 x BW) – (39.437 x females). The overall regression was not statistically significant (*r* ^2^ = 0.127, F (3, 21) = 1.02, p = 0.403). No predictors of Tmax at Follow-up were found.

The fitted regression model for t1/2 was: predicted t1/2 at Follow-up = 4.824 – (0.022 x age) – (0.062 x BMI) – (0.310 x females). The overall regression was not statistically significant (*r* ^2^ = 0.150, F (3, 18) = 1.06, p = 0.393). No predictors of t1/2 at Follow-up were found.

Considering BW rather than BMI, the fitted regression model for t1/2 was: predicted t1/2 at Follow-up = 4.455 – (0.020 x age) – (0.018 x BW) – (0.491 x females). The overall regression was not statistically significant (*r* ^2^ = 0.118, F (3, 18) = 0.80, p = 0.509). No predictors of t1/2 at Follow-up were found.

These results confirmed the existence of sex differences affecting AUC and Cmax, even after adjusting for other confounders such as age, and BMI or BW.

**FIGURE 3:**
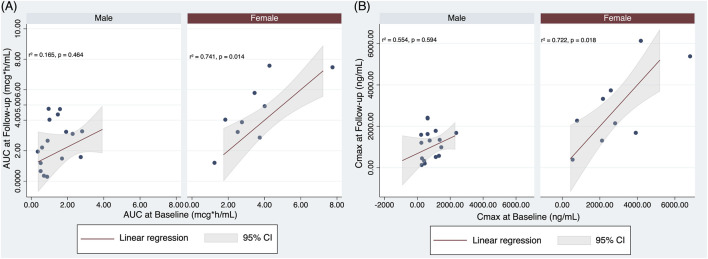
Linear regression analysis with **(A)** AUC at Follow-up as dependent variable and AUC at baseline, age, and BMI as independent variables, stratified by sex; **(B)** Cmax at Follow-up as dependent variable and Cmax at baseline, age, and BMI as independent variables, stratified by sex.

Moreover, to evaluate the possible correlation between PK parameters at baseline and Follow-up, linear regression analyses were performed, including age and BMI as confounders. BW was non tested, given the very similar result compared to BMI in the multivariate regression analyses. As shown in Figure 3 panel A, a positive significant correlation was found between AUC at baseline and AUC at Follow-up in females (beta = 0.993; 95% CI = 0.302 1.684, *r* ^2^ = 0.741, p = 0.014), but not in males (beta = 0.422; 95% CI = −0.793 1.636, *r* ^2^ = 0.165, p = 0.464).

A similar finding was observed on the Cmax (females, beta = 0.853; 95% CI = 0.221 1.484, *r* ^2^ = 0.722, p = 0.018; males, beta = 0.197; 95% CI = −0.586 0.980, *r* ^2^ = 0.154, p = 0.594), suggesting that females maintain the same LD PK pattern after 2 years of therapy (Figure 3, panel B).

At Follow-up, DYS were found in 20% of females but were not observed in males. Specifically, at Follow-up, 90% of females experienced WO as compared to 50% of males (Fisher’s exact test p = 0.022), with the only female without WO presenting the lowest AUC of all the others.

**FIGURE 4:**
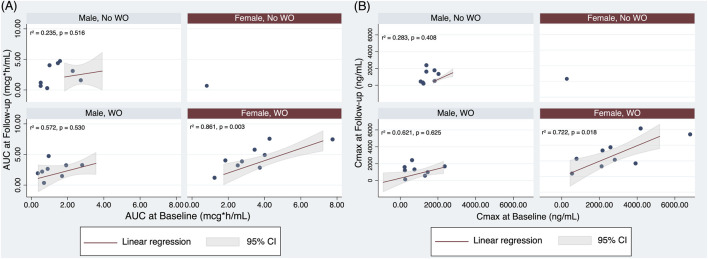
Linear regression analysis with **(A)** AUC at Follow-up as dependent variable and the AUC at baseline, age, and BMI as independent variables, stratified by sex and Wearing Off; **(B)** Cmax at Follow-up as dependent variable and Cmax at baseline, age, and BMI as independent variables, stratified by sex and Wearing Off.

A linear regression analysis, using AUC at Follow-up as the dependent variable and BMI, age, and AUC at baseline as independent variables, stratified by sex and presence of WO, showed that AUC at baseline was the best predictor of AUC at Follow-up in females with WO (p = 0.003, Figure 4 panel A).

The fitted regression model for AUC in males without WO was: predicted AUC at Follow-up = 23.624 – (0.245x age) – (0.230 x BMI). The overall regression was not statistically significant (r2 = 0.235, F (3, 4) = 0.41, p = 0.755). No predictors of AUC at Follow-up in males without WO were found (Figure 4 panel A).

The fitted regression model for AUC in males with WO was: predicted AUC at Follow-up = 7.882 + (0.104 x age) – (0.391 x BMI). The overall regression was not statistically significant (r2 = 0.572, F (3, 4) = 1.78, p = 0.290). No predictors of AUC at Follow-up in males with WO were found (Figure 4 panel A).

Only 1 female did not present WO. In this case, no linear regression was performed for insufficient observations.

The fitted regression model for AUC in females with WO was: predicted AUC at Follow-up = −4.938+ (0.071 x age) + (0.053 x BMI). The overall regression was statistically significant (r2 = 0.861, F (3, 5) = 10.29, p = 0.0140). The best predictor of AUC at Follow-up in females with WO was represented by AUC at baseline (beta = 1.059, 95% CI 0.559 1.559, p = 0.003, Figure 4 panel A).

Similarly, Cmax at baseline was the best predictor (p = 0.018) of Cmax at Follow-up in females with WO (Figure 4 panel B).

The fitted regression model for Cmax in males without WO was: predicted Cmax at Follow-up = 8814.306 – (74.786 x age) – (129.317 x BMI) + (0.823 x Cmax). The overall regression was not statistically significant (r2 = 0.283, F (3, 4) = 0.53, p = 0.687). No predictors of Cmax at Follow-up in males without WO were found (Figure 4 panel B).

The fitted regression model for Cmax in males with WO was: predicted Cmax at Follow-up = −6472.344 + (54.366 x age) + (153.647 x BMI) + (0.323 x Cmax). The overall regression was not statistically significant (r2 = 0.572, F (3, 4) = 1.78, p = 0.290). No predictors of Cmax at Follow-up in males with WO were found (Figure 4 panel B).

Only 1 female did not present WO. In this case, no linear regression was performed for insufficient observations.

The fitted regression model for Cmax in females with WO was: predicted Cmax at Follow-up = −11098.44 + (−11098.44 x age) + (192.286 x BMI) + (0.878 x Cmax). The overall regression was statistically significant (r2 = 0.769, F (3, 5) = 5.56, p = 0.048). The best predictor of Cmax at Follow-up in females with WO was represented by Cmax at baseline (beta = 0.877, 95% CI 0.300 1.456, p = 0.011, Figure 4 panel B).

Interestingly, in males with or without WO, no association was found between each parameter at baseline and Follow-up (Figure 4).

## 4 Discussion

As the long-term management of LD in PD patients is very difficult, it is crucial to identify biomarkers and clinical variables involved in therapeutic response and LD-related complications.

Sex differences in response to LD treatment have long been recognized and show that women, who are particularly prone to develop LD-related motor complications (Santos-García et al., 2023), have higher drug exposure than men (Contin et al., 2022; Conti et al., 2022; Kumagai et al., 2014).

In the earliest available studies evaluating the effects of oral LD in PD patients, it was not specified whether women were involved (Godwin-Austen et al., 1971; Stern et al., 1972), even when a critical plasma level was found above which dyskinetic movements became important (Reid et al., 1972). After the introduction of peripheral DOPA-decarboxylase inhibitors (DDCIs), benserazide and carbidopa, no sex-disaggregated data were reported in studies enrolling both men and women (Marsden et al., 1973; Rinne et al., 1975) and, up to now, women with PD are still under-represented in clinical trials, thus preventing clarification of biological differences based on sex (Smilowska et al., 2025). Plasma concentrations are strongly correlated with LD effects (Nutt, 2008; Murata et al., 1996), but unfortunately most studies that have investigated the relationship between LD PK and LD-related complications are retrospective or focused on patients treated chronically with different LD formulations and dosages^10,11,25^, making it impossible to translate the results into clinical practice.

In the largest retrospective study available in the literature, Contin et al. reported that the LD AUC was 27% higher in women than in men, concluding that female sex was the most important determinant of LD bioavailability. Age, weight and type of DCI also influenced drug absorption (Contin et al., 2022). Similarly, a recent retrospective study found that LD AUC in women was approximately 21.9% higher than in men. Furthermore, a 10-year increase in age resulted in an approximately 11.9% increase in the LD AUC. The study did not observe significant differences between sexes in the PK of DDCI (Miyaue and Nagai, 2025).

Our prospective data confirm these results, showing an even greater difference after 2 years of LD treatment than that retrospectively found by Contin et al. after a mean LD treatment time of 3.4 years. Furthermore, as we enrolled LD-naïve patients with no differences in terms of age, disease duration, and daily LD dosage, we suggest that sex differences in LD PK may occur independently of these factors (Conti et al., 2022).

We showed that LD concentrations, which were higher in women than men at baseline, remained higher in women than men at Follow-up. Interestingly, the PK curve drastically decreased between 40 and 60 min in women but not in men, as demonstrated by the slope of −57.94 ng·mL^−1^·min^−1^ measured in women and +9.26 ng·mL^−1^·min^−1^ in men.

Conversely, the effect of sex on LD PK was statistically significant for AUC and Cmax but not for Tmax and t1/2. In particular, the mean AUC and Cmax in women were higher than those in men by 2.130 mcg*h/mL and 366.966 ng/mL, respectively. It is important to note that this effect was independent of the time (baseline or Follow-up) at which PK analysis was performed.

We found that 2 years after LD introduction, 20% (n. 2) of women had DYS, while no men experienced this complication. In their large retrospective study, Contin et al. found that higher AUC and Cmax of LD were associated with an increased risk of DYS^10^. It has been repeatedly found that levodopa daily dose is an important predictor for dyskinesia in PD (Warren Olanow et al., 2013; Grandas et al., 1999). Unfortunately, due to the limited sample size, we were unable to assess any correlation between DYS and PK parameters.

In addition, Contin et al. found a higher AUC and Cmax of LD to be associated with more time spent in the OFF state, recognizing the latter result as unexpected, since higher LD concentrations should be associated with a reduction in the OFF time (Contin et al., 2022). Indeed, the relationship between increased time spent in OFF and WO episodes with increased LD uptake is difficult to explain. Many factors have been proposed, such as the changes in activity of metabolizing enzymes, gastrointestinal absorption, delayed gastric emptying, and the progressive degeneration of dopaminergic neurons (Leta et al., 2023).

It is largely recognized that, due to LD short half-life and variable gastrointestinal absorption, the way to reduce motor complications is to ensure more continuous LD brain delivery (Olanow et al., 2006).

A relevant finding of our longitudinal study is that all enrolled women experienced WO phenomenon, except one, who presented significantly lower AUC and Cmax values than the others. Notably, in women but not in men, the presence of WO episodes was correlated with AUC and Cmax at baseline and after 2 years of treatment. Furthermore, women showed not only a higher Cmax than men, but also an earlier peak in LD concentration followed by a drastic decrease in concentrations. It has been reported that this aspect is crucial in predicting the timeliness of LD Cmax because the speed of LD response reflects the rapid rise and fall of LD concentrations (Contin and Martinelli, 2010).

Our data are consistent with the results of some studies reporting that changes in the PK of LD undoubtedly contribute to the WO phenomenon. In particular, in contrast to the previous literature (Fabbrini et al., 1987; Gancher et al., 1987), Murata et al. suggested that the onset of WO correlated with failing LD plasma levels. However, although the study population was well balanced for sex, no disaggregated results were presented (Murata et al., 1996). More recently, Warren Olanow et al. reported that the risk of WO in LD-naïve patients increased with increasing LD dosages as for DYS, identifying young age, high UPDRS Part II, and female sex as predictors of both DYS and WO (Warren Olanow et al., 2013).

The present study has strengths and limitations. The strength is the prospective study design and the enrolment of patients naïve to LD treatment. On the other hand, the sample is limited, and further efforts are needed to expand the number of patients and observation time. On the other hand, Using the mixed–effect model, treating PK parameters as a random effect, the study accounted for the variability due to chance, such as differences between sampled subjects. This approach enhances the generalizability of the results to a larger population. Another limitation is the use of different DDCI (benserazide or carbidopa) at Follow-up, which prevented us from ruling out with certainty any influence on LD absorption.

Women with PD have higher plasma exposure to LD and are particularly prone to developing complications during chronic LD therapy.

This study examined whether sex differences in the pharmacokinetics of LD are associated with complications of chronic therapy with this drug.

We found that women are more exposed to the drug both at the time of first administration and when long-term therapy has been established. This aspect underlines the importance of developing sex-oriented therapeutic recommendations to be applied since the early stages of PD.

The identification of important differences in PK patterns between men and women 20–60 min after LD intake could be helpful both in planning further studies and in setting up a therapeutic drug monitoring. Indeed, as LD-related motor complications are difficult to predict clinically, the measurement of plasma LD levels may be very helpful in establishing the relationship between LD concentrations and clinical outcomes from both an efficacy and safety perspective, in order to optimize drug dosing from the start of treatment and possibly prevent LD-related complications.

## Data Availability

The original contributions presented in the study are included in the article/supplementary material, further inquiries can be directed to the corresponding author.
